# Supported Housing as a recovery option for long-stay patients with severe mental illness in a psychiatric hospital in South India: Learning from an innovative de-hospitalization process

**DOI:** 10.1371/journal.pone.0230074

**Published:** 2020-04-09

**Authors:** Archana Padmakar, Emma Emily de Wit, Sagaya Mary, Eline Regeer, Joske Bunders-Aelen, Barbara Regeer

**Affiliations:** 1 The Banyan Academy of Leadership in Mental Health (BALM), Chennai, India; 2 Athena Institute, Faculty of Science, Vrije University, Amsterdam, Netherlands; 3 Altrecht, Mental Health Care, Utrecht, Netherlands; ESIC Medical College & PGIMSR, INDIA

## Abstract

Individuals with severe mental illness have long been segregated from living in communities and participating in socio- cultural life. In recent years, owing to progressive legislations and declarations (in India and globally), there has been a growing movement towards promoting social inclusion and community participation, with emphasis on the need to develop alternative and inclusive care paradigms for persons with severe mental illness. However, transitions from inpatient care to community settings is a complex process involving implications at multiple levels involving diverse stakeholders such as mental health service users, care providers, local communities and policy makers. This article studies how the transition from a hospital setting to a community-based recovery model for personals with severe mental illness can be facilitated. It reflects on the innovative process of creating a Supported Housing model in South India, where 11 MH Service users transitioned from a psychiatric ECRC to independent living facilities. Experiences in various phases of the project development, including care provider- and community level responses and feedback were scrutinised to understand the strategies that were employed in enabling the transition. Qualitative methods (including in-depth interviews and naturalistic observations) were used with residents and staff members to explore the challenges they encountered in stabilizing the model, as well as the psychosocial benefits experienced by residents in the last phase. These were complemented with a Brief Psychiatric Rating Scale (BPRS) and WHO Quality of Life scale to compare baseline and post-assessment results and an increase of quality of life. Results display a significant reduction of psychiatric symptoms in patients (p< 0.5). It also describes the challenges encountered in the current context, and strategies that were used to respond and adapt the model to address these concerns effectively. Positive behavioural and psycho-emotional changes were observed amongst the residents, significant amongst those being enhanced in their mobility and participation. The article concludes by discussing the implications of this study for the development of innovative community-based models in wider contexts.

## Introduction

For many years, individuals with severe mental illness (SMI) were excluded from the community and confined in institutions. This isolation happened for various reasons, including, for example, ‘*a) the general attitude of the public about people with mental illness*, *b) a belief that the mentally ill could only be helped in such settings*, *and c) a lack of resources at the community level*’ [1]. With time, the institutional approach of segregation was much criticized during the 1950s and 1960s [2]. Institutions were criticized for functioning like warehouses, in which SMI were kept for long periods of time with no expectation of improvement or reintegration into the family and/or community [3].

In India, mental hospitals were introduced by the British as part of the psychiatric system, and as a way to purposely *‘segregate the mentally ill from the community and not treat them as normal but rather detention away from the community’* [4]. Mentally ill people were considered dangerous and were kept away from society [5]. In the 1970s, encouraged by the wave of de-institutionalization that had started in the West, development of psychiatric drugs, economic stagnation, lack of resources and gross understaffing problems, de-institutionalization took off in India, resulting in the decline of large psychiatric hospitals [4]. The process of de-institutionalization, defined as ‘…*the practice of caring for individuals in the community rather than in an institutional environment*’ [6], gave rise to more interest in community-based mental health care (CBMHC) for people with severe mental illness, as well as a widespread urgency to develop alternative models [2]. A new Mental Health Act in 1987 paid more attention to treatment and care, and mental hospitals were encouraged to function as active therapeutic centres, providing mental health services and community mental health.

The main argument advanced for this shift was that access to health care for people with longer-term mental disorders is much better addressed in community-based services than in the traditional psychiatric hospitals [7]. It would also be easier to promote the continuity of care and flexibility of services, making it possible to identify and provide timely treatment for relapses, and to increase adherence to treatment [7, 8]. The community-based services, when well embedded in professional mental health care, are also found to better protect human rights of people with mental disorders and prevent stigmatization [7]. Studies comparing community-based services with other models of care consistently also show significant better outcomes regarding adherence to treatment, clinical symptoms, quality of life, housing stability, and vocational rehabilitation [9, 10, 11]. Even so, with the positive outcomes of CBMHC models, there has been greater recognition of the need to approach innovative strategies from a broader cultural and institutional perspective, particularly taking into account the organizational and strategic processes that play a role in implementation of CBMHC models in India and in other low- and middle-income countries (LICs and MICs), and adopting various ways to dynamically implement and adapt to contextual factors in moving towards well-grounded community-based support.

## Community-based health models and Supported Housing (SH) in India

The state mental health system is the formal source of mental health care in India. However, due to poor resources and allocation of services, it is lacking in many ways [12, 13]. Less than 1 per cent of health expenditure is devoted to mental health care, in common with many LICs and MICs. As many mental hospitals still have large numbers of long-stay patients, leading to diminished recovery and reduced quality of life, community-based alternatives in mental health care are persistently advocated [12, 14].

**Fig 1:**
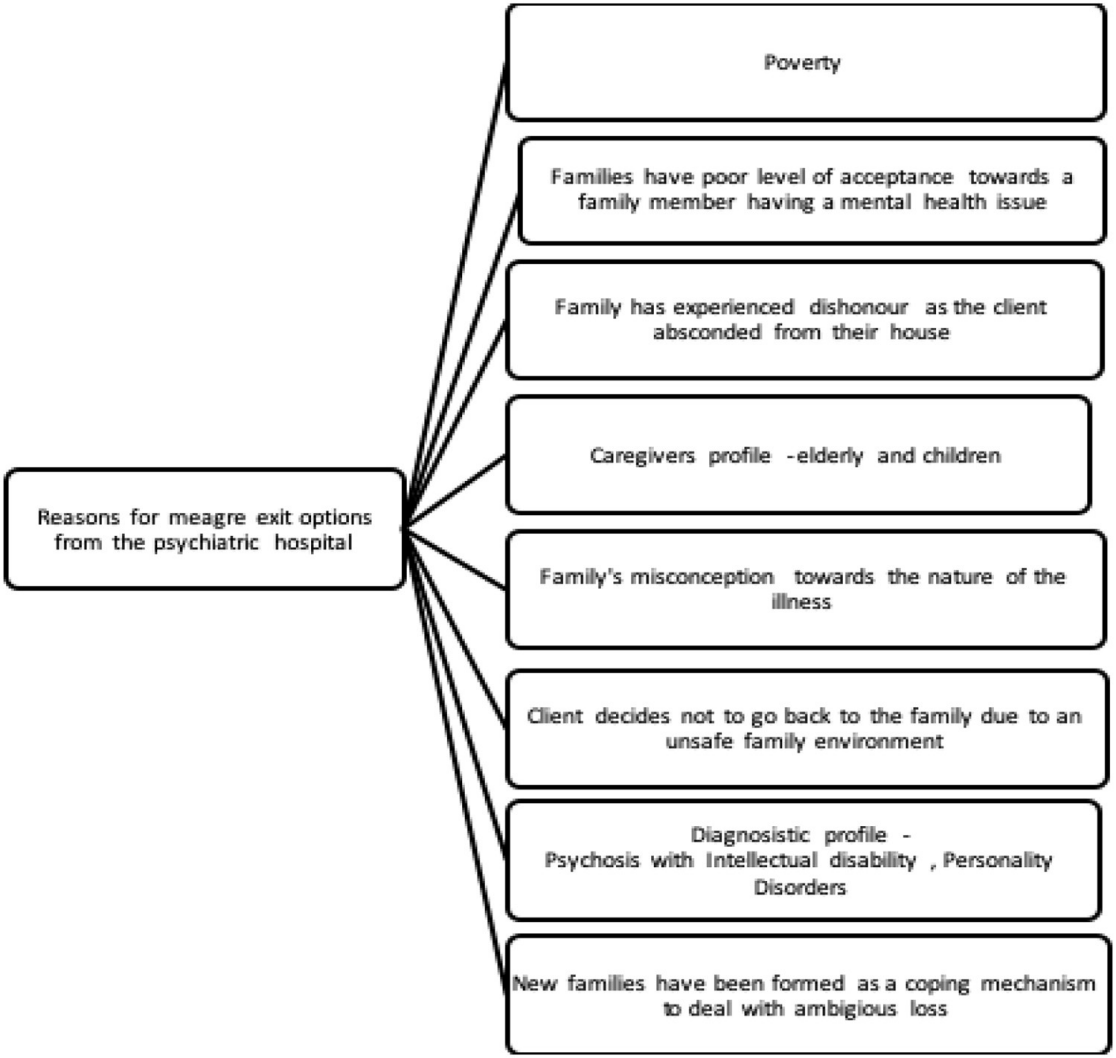
Reasons for prolonged duration in psychiatric hospitals.

In India, CBMHC commonly focuses on reintegrating persons with SMI into their family. Such an approach, however, denies support to a large population who have been abandoned by their family or, for many reasons, cannot return [15]. As there is a vicious cycle between mental illness, poverty, homelessness and stigma [16, 17, 18], many people remain excluded from long-term community care (see Fig 1). In most cases, homeless people are referred to homeless shelters or incarcerated in homes for beggars. Similarly, the commonly held belief that Indian culture is collectivist and therefore ready to support each other in distress is often not the case when stigma is involved [19].

Women are particularly vulnerable to being abandoned by their marital family, as ‘…*homelessness*, *vulnerability to sexual abuse and exposure to HIV and other infections contribute to the difficulties of rehabilitating women*’ [19]. It is essential to understand the challenges involved when there is no concept of ‘home’ to such a large population. Few existing programmes support those who have lost their home and therefore their identity in a community setting [15, 20, 21]. Similarly, CBMHC will need to be broadened to provide person-centred services for people with diverse experiences and histories of, for instance, homelessness, stigmatization, or gender-based violence (GBV), and so on [22].

It is in this context that supported housing (SH) emerged as an alternative that could meet the needs of SMI in order to better focus on functional impairment, social relationships, daily living skills and to promote recovery and self-reliance [23]. Supported housing provides structured, non-institutional, and independent living arrangements along with supportive services aimed at providing medical attention, rehabilitation and the attainment of life skills [24, 25]. These projects enable people to lead a normal and somewhat independent life, with support, ultimately aimed at rehabilitation and social reintegration [24]. Supported housing projects are user-centred, and place a strong emphasis on the residents’ independence, freedom of choice, independent life skills, individualized services, and ultimately community integration [26, 27]. Several studies have shown that homeless people with mental disorders who take part in a supported housing initiative show greater housing stability, make less use of shelters, are less often admitted and stay for less time in hospital, are less often imprisoned, and less substance abuse [27]. Since this model is not often described in the context of India, this study aims to understand how moving towards SH may support people with severe SMI. More specifically, it hopes both to shed light on how SH functions in a specific context in India, and also how to develop the concept of SH in a sustainable, sensible matter in the broader context of de-hospitalization.

### The banyan’s supported housing model

The Banyan is an organization that provides comprehensive mental health services in institutional and community settings for people experiencing poverty and homelessness [for a full description of the evolution of The Banyan, see [28]. Starting in 1993 with a crisis intervention and rehabilitation centre for homeless women with mental illness in the city of Chennai, The Banyan has grown to provide wellbeing-oriented mental health services, including emergency, open shelter and street-based services, long-term and alternative living options, and social care. Some 20 years ago, The Banyan also began to focus on catering to people as part of the community, shifting from long-standing institutional care to a model of community-based care. Since 1993, The Banyan has supported 1,691 homeless women with mental illness: 1,065 have been reunited with their family or community of origin; and 80 women access SH through a housing-style open facility in the community. Almost 2,500 have attended community clinics and associated social care services (such as livelihood and welfare facilitation in rural areas and towns).

**Fig 2:**
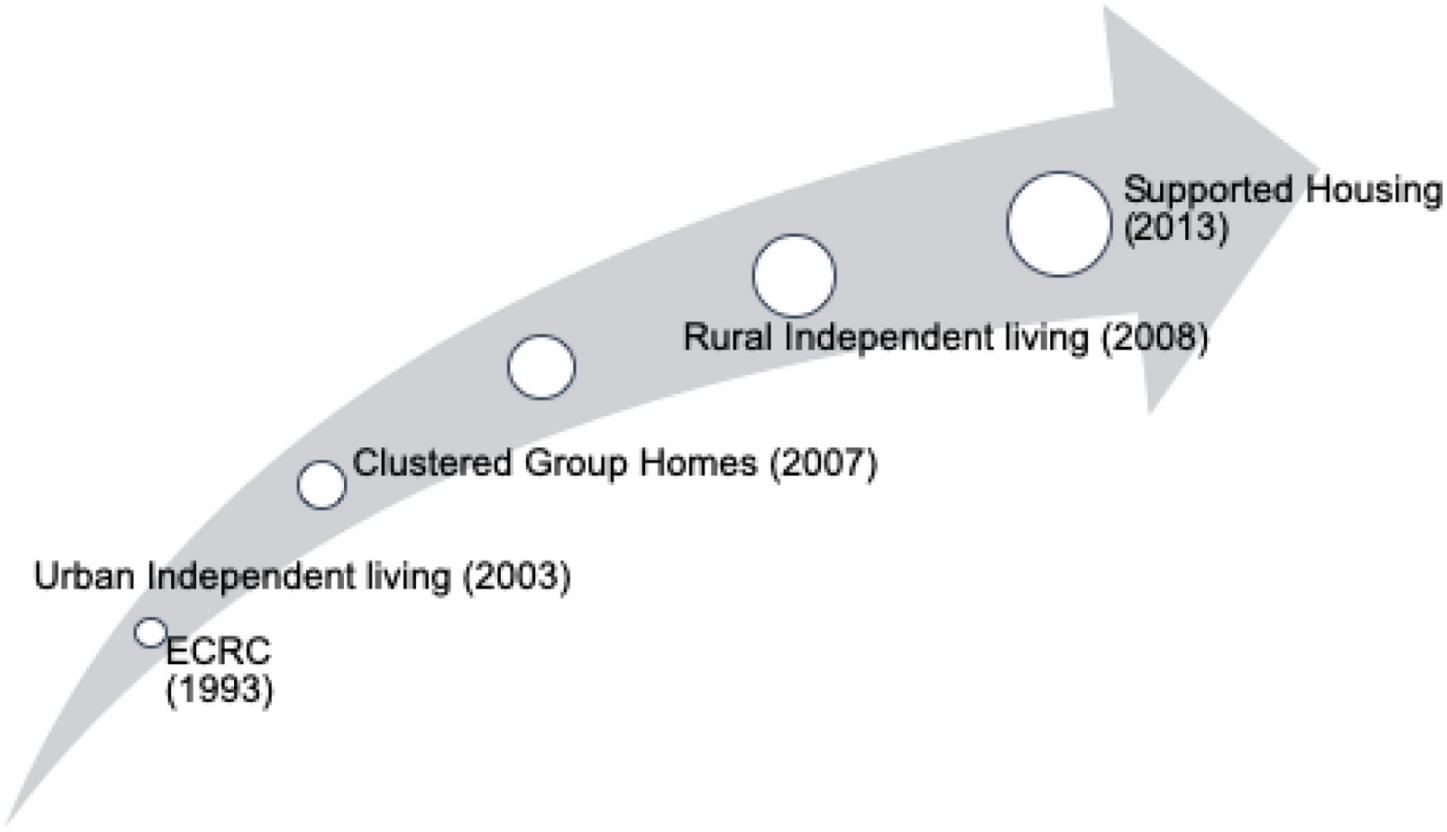
History of The Banyan working towards supported housing.

The Banyan has explored housing options at different stages in its trajectory as a response to the complexities in mental health services. It started its first housing options for homeless women in 2003, intended exclusively for women who were completely functional and recovered. In 2007, long-term residents were moved to a quasi-institutional living arrangement situated at a Banyan facility. Independent living in the rural location was initiated in 2008 and was conceptually similar to the urban project. In hindsight, a systemic change can be observed in how after-care was organized for patients with chronic needs. Though institutional care has a range of services and support systems catered for in a user-centred environment, there was a strong need to understand and accelerate the slow progress of residents where negligible or no qualitative gains were seen in terms of personal recovery, and/or progress in participation. Accordingly, The Banyan undertook a pilot study with a supported housing facility near the Emergency Care and Recovery Centre (ECRC), where residents were selected and moved to live together with health care workers (HCWs) (see Fig 2).

### Study objective: Learning from niche experiments

To our knowledge, little has been written on how to move from an undesirable situation (e.g. prolonged hospitalization of chronically ill patients) to a community-embedded care model in India. Indeed, as described by Thornicroft [26] and Lewin [29] on CBMHC, the transition of people from long-stay wards to community-based care (particularly in LICs and MICs) often faces various challenges and obstacles, such as stigma and discrimination both in society and by primary health staff, old or inappropriate programmes and legislation, inadequate alignment between the intended services and outcomes, feasibility problems, poor uptake by various (non-) professional stakeholders and potential collaborations, high staff turnover and/or burnout, etc. In order to create models that foster sustainable community care (and deal with the complexities involved), there is a need to learn from pilot studies or *niche experiments* [30], in which new approaches to embed patients in a community setting are developed, practised and reflected upon. This study aims to contribute to filling this gap in *socially robust knowledge* [31] by describing the development of an SH project in India in the context of The Banyan’s practice.

To understand how transitional processes work, and what can be learned from them, several models have been proposed in the field of change management, including Lewin’s change model [29], Kotter’s theory of change [30], as well as more recent ones such as the Stages of Implementation Completion (SIC) model developed by Brown, Chamberlain & Saldana [31]. Managing change generally starts with a period of *‘unfreezing’* [32] or *‘pre-implementation*’ [31], including the development of urgency, vision and alignment. Consequently, a period of *‘change’* (implementation or empowering actions) commences, usually including both *confrontation* (frustration and anxiety) and *adaptation* (strategies to respond to barriers). As the change becomes increasingly integrated, a more *stable* phase of sustained change is reached (see all phases in Fig 2). Bridge’s Transition Model [32], finally, helps to emphasize the identity transitions (for instance of health workers and patients) that are involved in processes of change. According to Bridges [32], as transitions materialize, feelings of disorientation, fear, uncertainty and frustration may initially arise. People are in a process of (slowly) letting go of a particular practice and getting used to the idea of a new beginning. Particularly during the implementation phase, feelings of resentment or anxiety about the new roles may be involved, but also great creativity, innovation and new ways of thinking about dealing with the situation (strategies). The final stage is a time of acceptance and new energy, where all stakeholders have embraced the change, and built on the skills that were required to make the new model work [32].

**Fig 3:**
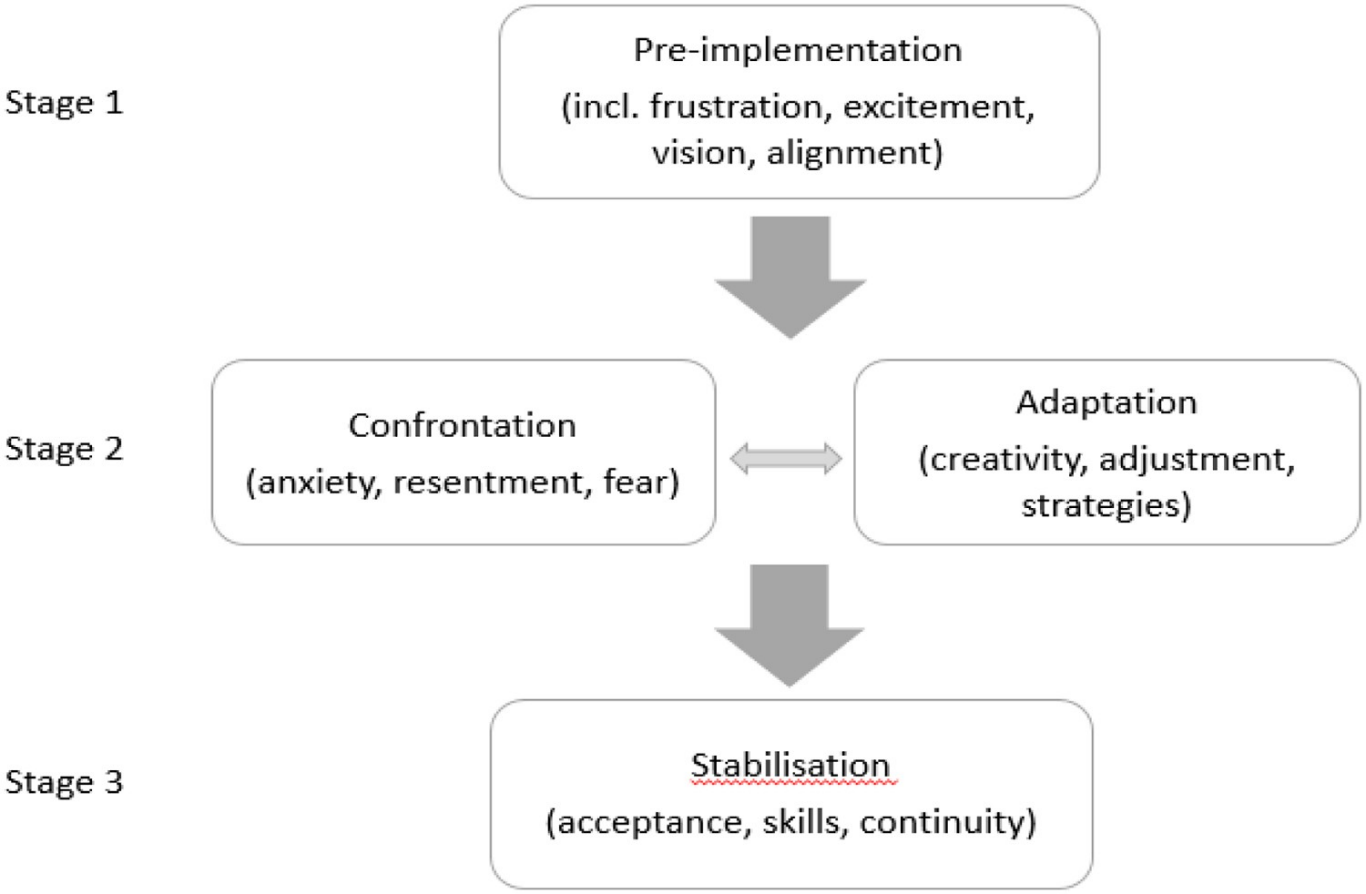
A model to evaluate the three stages of transition towards supported housing.

In Fig 3, the earlier theories are combined to visualize how three stages in the change process towards community-based mental health (or more specifically, SH) can be followed to understand the transition process. In documenting how residents and staff perceive the current approach of community-based mental health care, it is important to consider all sides of the experience [33], starting with residents’ initial transitions into housing. This is especially true, as the experience of transition can be stressful for all concerned, especially for residents who have a history of homelessness, the revolving-door syndrome, and continuous psychiatric health problems.

### Methodology

This study included mixed methods. In order to understand how The Banyan worked towards establishing the SH housing project, qualitative data was drawn from various sources at different phases of the project. In order to understand the process of shifting and recovery from ECRC towards supported housing, the following questions were addressed:

1. How did the selection process of residents, house and HCWs take place? What were the steps involved in the pre-implementation phase? (Stage 1: pre-implementation)
2. What challenges were faced by the residents and the HCWs after moving into the supported housing? And, what strategies were employed to deal with the challenges? (Stage 2: confrontation and adaptation)
3. What were the components of recovery that were observed in the residents after moving to the shared house? (Stage 3: stabilization)

A large part of the data was collected throughout the development of the project, such as observational notes and daily log book records, mostly produced by HCWs and case managers in the SH setting. Great care was taken to report systematically on residents’ activities of daily living, interactions, non-verbal communications, etc. (hereafter referred to as residents of SH). Log books were maintained by the HCWs in the SH, and staffs would visit and record accordingly. Visitors and other colleagues were encouraged to write in their comments for feedback and also reflected on areas to improve. Information contained in the log books also gave feedback regarding the structure and working pace of each individual. Significant incidents and behaviour were thus recorded. Also, Appreciative Inquiry (AI) conversations were also held with 11 SH residents and noted by the HCWs. Topics included in the conversations with patients are displayed in the interview guide (see S1 Appendix).

Before and after the project, In-Depth Interviews (IDIs) were held with 14 members of staff involved in SH, including: healthcare workers, community workers, case managers, project managers, and management members. The individual interviews were conducted to understand how the different stakeholders experienced the transition from ECRC towards SH in The Banyan. Using a timeline interview format, the participants were asked questions related to the definition of SH, the development of the concept, various SH practices, as well as the challenges they encountered and successes they achieved. In addition, the timelines were used to identify and characterize the different strategies used to improve the SH project and stabilize its positive outcomes. Such interviews, semi-structured as timeline histories, are useful to identify the sequence of events and the rationale behind this development, as well as the experiences and perceptions of the people involved [34]. As more data was collected, the interview questions became more specific, and more attention was given to the support of the organization to facilitate the SH, as well as to the identification of crucial aspects in the SH approach.

The interviews and log books were conducted in Tamil and transcribed verbatim. Tables were made in comparing observations between clients while they were at ECRC and while they were in SH. Three independent researchers worked on analysing the data, first by reading the transcripts and the notes. Tables were drawn for comparison and then the data was compared and clustered according to daily function categories., then performing open coding to derive themes and sub-themes. The researchers began the bottom-up phase by reading the transcribed interviews carefully and identifying relevant text fragments, i.e. words and phrases that were meaningful to the research focus. Each new perspective on recovery of mental health was given a new code. Text fragments which expressed a common idea were grouped into categories. For example, categories such as ‘feeling happy’ and ‘feeling connected as a family’ reflected emotional and social aspects of wellbeing respectively. Systematic comparison helped to identify associated categories [35]. The categories were then organized according to the four phases of implementation. The authors and a research assistant conducted the analysis in regular meetings to check each other’s assumptions and resolve disagreements before writing up the results.

#### Quantitative measurements

Finally, the qualitative data was complemented with some clinical measurements, such as the World Health Organization Quality of Life scale (WHOQOL) to measure four conceptual domains of the quality of life, including: Physical Health, Psychological, Social Relationships and Environment. The measurement was administered as baseline and post-assessment, as well as three intermittent measurements (three in total), with 11 residents. The outcomes on this scale were evaluated by performing a paired t-test in SPSS, to detect any significant differences between the baseline and final measurements, using a probability of p < .05. Similarly, the Brief Psychiatric Rating Scale (BPRS) was used to compare before- and after scores in patients, to see if there were any changes in psychiatric symptoms, including anxiety, depression and unusual behaviour [36]. The outcomes on this scale were also calculated with a paired t-test in SPSS, using a probability of p. < .05. Both the WHOQOL and BPRS was administered by a Psychiatrist and the WHOQOL was administered by either Social workers or Psychiatrists.

#### Ethics

The study proposal was reviewed and approved by The Banyan’s Research Review Board (RRB). Participants were informed about the purpose of research and of their right to confidentiality and to refuse to respond to any or all questionnaire items. Informed consent was obtained from all participants prior to the interview, and personally identifiable names or details were removed. Care was taken to inform all the participants in detail about both the intervention and research process, and their right to withdraw from the study at any moment.

## Results

In order to move towards realizing the SH concept, The Banyan took a number of preparatory steps. Although the concept of SH was quite well developed in theory, the way this vision would be shared with and for whom it would be implemented, and how the different ideas and challenges regarding the realization of SH in the given context were to be negotiated, was learned during the process. Second, as the team moved to implement the concept, various activities helped to mediate the challenges in the confrontation phase, and to facilitate successful adaptation. The analysis of interviews with health workers and residents, as well as the naturalistic observations and field notes, give insights into what came up during these phases.

### Stage 1: Pre-implementation

#### Selection of residents

Stage 1 started with selecting those who would be eligible to move to the SH facility and would thus be offered this option. Eligibility was based on two main criteria: first, they needed to be living in the ECRC for a period of five years or more, and second, they needed to have shown limited improvement in their mental illness over a prolonged period of time. The Banyan already had a decade of experience with independent living arrangements for high-functioning residents and few possibilities for reintegration. This pilot study focused on those with chronic needs and who required a high level of HCW support. The team looked at clients who had little to no possibility of reintegration with their family and community. One HCW reflects on the selection process:

*We took residents who were really not doing well*. *Some residents could not even take a bath by themselves*. *We took residents like that to think about supported housing*.

A project manager reflects on the complexity of profile of residents as follows:

*These were people with chronic symptoms*. *They showed less progress*, *may be due to prolonged periods of hospitalization*. *[You need to] understand the profile*, *it’s a complex category*.

Fifty-nine of the 110 individuals who were initially identified as meeting both criteria (i.e. they had lived in the ECRC for five years or more and had not shown much progress). The mental health status of this group of people was evaluated using the BPRS, and scores higher than at least 41 (moderately ill) were considered. Most clients had scores of over 50, which is considered markedly ill. The total final number of participants was 11 patients (M = 50.3, SD = 14). After staying in the Shared Housing facility for five months uninterruptedly, the average BPRS scores of patients dropped to an average of 34.7, which was found to be a significant reduction (t = 2.46, p = .0233).

As a way to discuss the SH concept with mentally ill residents, the staff decided to work with drawings and word associations, and stimulate the discussion from here. The staff member would draw an independently standing house, with windows, a living room, and separate bedrooms, and ask the residents what this picture meant to them. The reactions were varied. Some said: ‘*this is my home*’ or *‘this is ECRC’*. Others said it was family, or that they had lost their home. Through this bottom-up approach, the staff members were able to explain what the SH concept would look like and that some of them perhaps would like to move there. Some were immediately opposed to the idea, and felt the risk of being abandoned. One resident would ask: ‘*don’t you like me anymore*?*’*. But others felt open to thinking about the idea, especially as it could create more silence and privacy for them. As one resident said: *‘In ECRC I have to share my food plate and sometimes my stuff gets stolen*, *and then I have to find it*. *I am too old for such things’*.

As a next step, interviews with each of the eligible residents were conducted to find out about their preferences, specifically with regard to living with four other housemates and their living arrangements.

*We asked them how they would want to live*, *what kind of food they want to eat*, *what kind of clothes*, *what are their work pursuits*, *what are their interests*. *And we also informed them about the supported housing option and asked them whether they would like this kind of choice*.

Pre-engagement sessions were organized to discuss and negotiate the potential changes this initiative might bring and what challenges they might face. The sessions were also used to discuss what living in a supported house entailed and to encourage them to discuss any questions they might have with staff, case managers and among themselves. The respective case managers also had a debriefing meeting with them as necessary. As a case manager reflects:

*It was very direct and open conversation*, *asking a client about the shift*, *who would you like to stay with*. *And then they would come back with their ideas saying; ‘this is what I would want to do’*. *The dialogues were very open*, *in terms of what fears they might have*, *how they would want to live*, *how much personal space they want*, *how would they make this home life for themselves*. *So other things were also discussed other than just structural relocation*.

Out of the 59, eventually 20 residents agreed to move out to the SH facility. Again, they were asked about their preferences and especially with regard to their choice of housemates.

*They would need to have something in common*, *it could be affiliation*, *familiarity*, *age*, *language and regionality*, *it could be based on preference and shared interests*. *Whatever the connecting component might be*, *they all decided whom they would share their home with*. *There was no coercion*. *They would all say*, *‘yeah*, *I would like to stay with them*, *I would be happy there’*. *So there was some kind of affinity*, *camaraderie*, *friendship that determined who will stay with whom*.

#### Selection of the house

Finding a house for residents to live in a community was the biggest challenge faced by The Banyan, but an important step. Property owners were unfamiliar and uncomfortable with the notion of SH for the mentally ill. The process of finding the first house took about four months, because of some of the landlords’ dogmatic attitudes towards renting out their house to the organization, and the social stigma related to mental illness. They argued that they would fail to find future tenants if people knew that residents with mental illness had lived in the house. House owners also believed that people with mental health issues need to be locked up in homes or hospitals. Moving them out into the community was considered too risky.

Property owners had a preference for functional and mobile residents. Some landlords also had difficulty in accepting that persons with mental health issues can live as families within a community setting.

One property owner was finally prepared to support the approach and decided to let out his house. The project manager remembers the conversation with him (see Box 1).

**Box 1. Reflection of a project manager concerning the house**

[…] so this was one particular gentleman who knew The Banyan’s work, and he also struggled with the concept in the beginning. I [project manager] had explained this concept to him […], so he didn’t understand the fact like, why are you moving out people, why not keep them here? […] I had another admin person next to me, so we said we are doing this as a trial […], we want to work this option out, and see if it is going to work for residents first, and secondly for us. So, he said, it is a good initiative, at least you are thinking out of the box. […] he understood what it was and, he also asked for few terms as in, you shouldn’t make too much noise, you should keep the house clean, which is very normal things to ask […] then we said, can we have an extra bathroom and all that, and then he was very willing to help us with that. So, he took us around […], he did it in a very nice way, that we felt it was a nice place, […] we felt it was safe, so we saw the infrastructure first, we were convinced that, okay, this is a good place to live in, that’s when we started speaking, […] to residents as well.

#### Selection of the health care workers

The SH arrangement required two HCWs to work in shifts in one house for five residents. Individual and group sessions were done with The Banyan HCWS to discuss their change of role and they were given time to decide if they wanted to work in this new setting.

The training session included information on the components of the SH model as an alternative approach to care and the innovative role of HCWs. Also, before accepting to live in the SH property, the HCWs visited the house in order to get acquainted with the residents who would be moving there with them.

Two HCWs volunteered and were willing to commit to the concept. Despite not being familiar with the idea of supported housing, they were enthusiastic to explore how it would work. The other HCWs preferred not to move for different reasons, the main one being that if they lived in the SH facility, they might not be able to see their colleagues and friends frequently (see Table 1).

**Table 1:** Overview of factors that enabled the selection process.

With residents	With house owners	With HCW’s
Systematic process to identify the potential population who require long-term support Creative and accessible way of explaining the housing model Opportunity to make a preference list (people, housing arrangement) 4. Opportunity to take time to think and take a decision Provide an environment that accommodates changes in decision regarding going back to ECRC	Finding a property owner who is aware of the organization’s work Finding someone who understands the need for innovation at ECRC Accommodating to property owners’ concerns	Debriefing on the new role Training sessions for the HCWs Visit to the supported housing Address challenges by brainstorming strategies to resolve any issues

### Stage 2: Confrontation and adaptation

#### Residents encountering confrontation and adapting to the new situation

According to HCWs, particularly in the first period after the move towards supported housing, there were many transitions back and forth between the SH and ECRC. Some residents were ambivalent about moving into the supported housing and moved in and out, in some cases multiple times within the span of six months. For some, the process of final transition took three months, while others continued to stay and enjoyed having a space of their own and living in a less crowded environment. This eventually led to only 11 residents living in the supported housing.

These 11 residents were all women over the age of 40. Some eight women were married, one widowed, one divorced and the marital status of one was unknown. One of the residents had no formal education, one went to primary school, two to middle school, and seven to high school. Five of the women spoke Hindi, three of them Tamil, one Bengali, one Telugu and one Malayalam. Seven were diagnosed with schizophrenia, three with psychosis and one with a mood disorder with psychotic symptoms. Four of the residents had been hospitalized for six to 10 years, six of them for 11–15 years and one for 16–20 years (see Table 2).

**Table 2:** Socio-demographic characteristics sample (N = 11).

***Age (In years)***	
Mean Age	56
***Age range***	
40–49	3
50 and over	4
60 and over	4
***Sex***	
Women	11
***Marital status***	
Married	8
Divorced	1
Widowed	1
Unknown	1
***Duration of stay at the psychiatric hospital***	5 years and over

During the confrontation and adaptation phase, different reactions were observed among the residents. ‘Separation anxiety’ from a known environment was commonly seen, as one HCW reflects:

*They got a bit anxious in the new environment*. *Those residents who lived at ECRC for more than 10 years got worried in a new environment*.

A fear of ‘losing the familiar’ was noted by the CHWs, in response to which a supportive strategy was developed. Residents could visit or return to the ECRC whenever they wished. The close vicinity to the ECRC made it particularly easy to arrange the logistics. Residents were again reassured that they would be receiving the same care programme during their stay in the SH facility.

If residents had already moved but showed an unwillingness to stay in the SH facility, the HCWs addressed their concerns immediately and residents returned to the ECRC. This also made it clear to other residents that they had the choice of moving out and could come back again, which helped in reducing separation anxiety.

One interviewee described how residents could change their mind and decide to move back to the ECRC, a step that was not perceived as regressive or poor decision-making as an individual’s free choice:

*… [some] wanted to go back to the secure [safe environment] of the ECRC*, *not really because they were ill*, *but they just felt that they were*, *you know*, *ECRC is their home*. *We didn’t see it as regression into something*, *if that is where they feel they belong and they want to be*, *because they are otherwise well*, *and they have friends there*, *they like several things out over there*, *so we must give them the liberty to go back*, *and not see it as going back to institutional care*.

Though the ‘fear of losing the old identity’ among residents was prevalent, there were also themes regarding ‘fear of the new environment’. In the supported housing, it was less crowded and quieter. To make the residents familiar with the new environment and make clear that it would be a personal choice to move, The Banyan initially organized weekend trips to supported housing. Residents stayed at the SH facility during the weekends. As the SH facility was situated only a kilometre away from the ECRC, they felt a greater sense of safety and comfort.

As one of the HCWs reflects:

*I gave them lots of activity in a good way*. *We called a social worker who gave them orientation in Hindi*, *this is our home*. *We all can live together*, *so we all can love each other and be friendly*.

Despite the fact that the house was situated in a known locality, differences in the environment seemed insurmountable for some residents. Most residents had experienced abandonment by their family and were thus sensitive to relocating. This made it crucial for the staff to communicate clearly that SH is geared towards a recovery approach and that the transition could be reversed at any time. The residents were also comforted in their fear of being left behind through clear communication and arranging visits by their friends.

Staff working in the supported housing were encouraged to observe and directly address feelings of anxiety in the residents and to try to find solutions fit for each individual person. HCWs assisted in following the treatment plans developed for each resident living in the home (see Table 3).

**Table 3:** Challenges faced and strategies employed to address these (stage 2).

Challenges (confrontation)	Strategy (adaptation)
Fear of losing the ‘known’	Residents could visit ECRC whenever they liked, including for celebrations /festive days Residents can relocate to the ECRC at any time Residents are reassured about receiving similar care as at the ECRC HCWs assisted in following the treatment plan developed for each resident
Fear of abandonment	Regular visits by residents’ case managers and friends from the ECRC and clear communication addressing their autonomy and independent decision-making regarding the move
Fear of the ‘new’	Exploration of the environment by residents along with staff Constant reassurance by case managers and HCWs
Condition of resident	People with an intellectual disability found it particularly difficult and there was greater moving to and forth among them

#### Health care workers confronting and adapting to the new situation in stage 2

HCWs also experienced challenges in the process of adaptation (see Table 5). Only two HCWs were initially willing to take on the new role. There was a high sense of uncertainty and apprehension associated with the new role, and also with the community and social environment.

The core concept of integrating residents back into the community posed great challenge to HCWs. One HCW reflects how much she was overwhelmed during the initiation of the project:

*It was very difficult; I had to do most of the household work*, *the residents would help me at times*. *I also wanted to leave the facility at some point*. *But slowly there was improvement in residents*. *In the beginning*, *neighbours used to come and ask me why they are shouting and behaving in a different way*. *Once even*, *I cried because they could not understand my feelings*. *I told all the neighbours that residents here […] are in training to get back to their usual life*. *This training will make them independent*. *And they can live on their own without depending on others*. *So*, *I used to beg them ‘please understand’ and sometimes I felt like running away from supported housing*. *Some residents had disturbances in the night and had interpersonal issues with the neighbourhood*.

She continues by describing how things gradually changed after some time:

*Later on*, *I spoke to the residents*. *Even they are persons like us*, *not good to avoid them*, *try to talk to them*. *Also*, *I informed the neighbour about this model*. *Slowly residents started to socialize and visit neighbours’ place and started spending time together*.

Some HCWs expressed discomfort about sharing space with residents who would occasionally engage in hostile behaviour. This was aggravated by the fact that the HCWs could not ask colleagues for help as they would do in the ECRC, as only two of them lived and worked in the supported housing. As one HCW reflects:

*It’s a new environment; that could be the reason why residents got emotionally disturbed*. *I was alone and I was in charge of 2 x 5 residents*. *Then there was also an incident when a client assaulted me*.

Moreover, the HCWs felt much ambivalence about their new role in the SH model. As one interviewee reflects:

*Good care is not driven by a set of protocols*, *so there was a lot of confusion in the beginning among the health care workers; can we allow them to have social transactions*, *[…] what if they are not dressed well*, *you know*, *or not brushing their teeth*, *what if they don’t take their medication*, *what if they get lost when they are out in the community*, *those were challenges that we were tensed thinking about*, *but I think they have learned only by doing*.

To support the CHWs, capacity-building sessions were held, particularly focusing on how to provide care while empowering residents to live together independently. The training aimed to support HCWs in finding a balance between ‘protecting residents (from other residents and from themselves)’ and ‘stimulating their autonomy in a gradual manner’. One interviewee explains:

*And*, *of course*, *we had to*, *we couldn’t leave them [the residents] entirely on their own as they did still need a lot of support*. *So*, *we had HCWs*, *who were trained on care processes*, *purpose of exercising autonomy in a home setting*, *to go and do what they want to do*, *and yet be there as*, *like*, *you know*, *carers in a home setting*.

Another challenge that some of the HCWs experienced related to their changing role was their feeling that they had to explain or even justify living in the community as unmarried women. HCWs were reassured not only by creating safety measures and financial reimbursement, but also by offering extended support to explain to their families about their (new) professional role and associated responsibilities. This helped to make the HCWs feel more comfortable with their work.

Among the HCWs in the ECRC, there were some who felt that the SH work was less demanding and so should be reflected in a lower salary. As a strategy to deal with this demand, the intense nature of work in the supported housing and the change in care model was explained to all HCWs (see Table 4).

**Table 4:** Challenges faced by HCWs and strategies employed to address them.

Challenges (confrontation)	Strategy (adaptation)
Apprehension about the new job role and environment	Before accepting their new role, HCWs visited the supported housing Training was conducted and researchers were supportive to address any issues Support was provided to HCWs to make their own conscious decisions regarding their new role
Independently handling residents’ care and anxiety due to living with residents in a home setting	In individual and group sessions the HCWs were prepared; illustrations of residents’ behaviour and hypothetical situations were discussed
Difficulty in accepting the model as a form of transition towards community integration	Dissemination of information on the SH model as an alternative approach to care. Training components included: a) Innovative care model of supported housing–focus on preference, shared decision-making and HCWs as well as deliberately unstructured environment b) Aligning this care model to the strategy and vision of The Banyan, also bringing about staff changes in the organization c) Role of HCWs in supported housing
Families of HCWs had apprehensions about them living in a home setting	HCWs were assured of job security, pay scale and a job role equivalent to those who were working at the ECRC. Also, in certain situations the researchers intervened and convinced family members.
Sharing common space	This was the first time that living space was shared between residents and staff members in a home setting. Hence, certain challenges such as sorting out personal spaces in the rooms, roles and responsibilities in keeping the living space organized were prevalent and immediately dealt with by the HCWs.
Job role and description differs between hospital setting and supported housing	The nature of work in the supported housing and the shift in treatment was explained, and the intensity and the depth of the work were emphasized

### Stage 3: Stabilization phase

The stable phase describes the outcomes observed when most of the changes were integrated and the project required only maintenance. It is during this phase that some of the intended outcomes (e.g. better quality of life, independent living capacity, and lessening of symptoms) could be evaluated, and strategies to sustain successful change could be addressed.

Among the residents, several social and behavioural changes were witnessed, after a period of adjusting to the new environment. Positive changes at various levels were observed in the SH residents. Here, we present one resident, Ms M., to illustrate the developments that happened after moving to the supported housing in the stable phase.

Before Ms. M moved to the SH facility, she showed poor social skills, functionality, participation and was highly withdrawn. After three months of continuous stay in the supportive housing, she showed significant progress in every dimension. While at first Ms M. would keep to herself most of the time, she somehow gradually started socializing, such as making tea for her housemates and staff and going out for walks with others. She showed greater physical mobility, her self-care improved (also needing less assistance), sharing food, and even showing interest in cooking (see Table 5).

**Table 5:** Comparison of social and behavioural outcomes in Ms M.

No	INDICATORS	Observations during pre- and post-housing intervention	
		During stay at ECRC	After 6 months in supported housing
**1.**	*General Behaviour*	She prefers to sleep and lie down during the most of the day She was irritable	She continues to sleep and lie down during the most of the day She is less irritable She appears to be cheerful
**2.**	*Self-care*	She required several prompts to focus on self-care	She was able to take care of herself without any prompts and also started bathing on her own
**3.**	*Mobility*	She would refuse to get out of her bed to eat Ms M usually requests the HCW to serve her food and prefers to eat in bed	She would walk to the dining table to get her own plate and serve her own food
**4.**	*Daily Chores*	She required several prompts to work and to engage in any activity	She started showing interest in washing clothes, drying and folding them She started cooking by making chapati dough and makes tea in the evening for everyone at home Started watching television
**5.**	*Earning*	There was no means of payment	After several prompts she was able to engage in activities Engagement in social enterprise (selling rice batter) increased as it was yielding a profit
**6.**	*Social Skills*	She does not socialize spontaneously, only when someone tries to socialize with her Socializes in a minimal, monosyllabic manner	She speaks very politely to HCWs and to her housemates She also initiated very limited conversations with her daughter

Similar developments were observed in the other residents since moving into the SH facility. For the other residents, for instance, improvements in self-care were particularly noticeable, as some started to take a bath by themselves and wash their own clothes. Many such changes were manifested because residents felt more at home, and so also took better care of the house. It was no longer only the HCWs who were cleaning the house as some residents also did so. Some also started cooking meals and serving tea to each other and to their occasional guests. One resident had the habit of spitting, but she managed to spit into the sink rather than on the floor or elsewhere. This change happened after HCWs had explicitly mentioned to her that this is her space, i.e. her own home. The other major change relates to engaging economic activities as many women started to sell vegetables, flowers and different kinds of batter, in the vicinity. Most residents seemed more relaxed and generally expressed feeling better in a non-hospitalized environment.

Based on the HCWs’ notes, subtle changes were also seen in the roles that residents took on in their new environment. As one HCW reflects:

*I see certain changes when I visit these two houses*. *Earlier as I would work in the hospital*, *it would be*, *‘give me this*, *give me that’*, *no reaction*, *or just a smile*. *Whereas over here*, *I receive a smile*, *but it is a very encouraging smile and they have started communicating in simple ways by saying; ‘Can I make you some coffee/tea*? *‘Have you eaten your breakfast*?*’ You can see how they are playing the role of provider whereas earlier they would be very passive in the hospital setting*. *There is a change in identity in my opinion; very uplifting and very indicative of hope*.

Naturally, a culture of ‘family’ and a simulation of a ‘household’ was developed. It was observed that there was more acceptance of each other and residents were also more likely to perceive HCWs as family members. As two HCWs mentioned:

*Each of the houses had the same philosophy; let’s try and weave in a thread of what a family stands for*. *Typically*, *the resident would refer to the HCW as [very few would refer to them as staff] my daughter*, *my daughter-in-law*, *which actually here is good as it fosters a bonding with the HCW which are typically familiar relationships in many ways*.

*In supported housing we all sit together and have our food while in ECRC*, *the residents and workers will have to eat separately*.

Gradually, the quality of life for residents in the SH project seemed to mirror that of a home-like environment. Such improvements were also reflected in the quantitative results. Outcomes on the WHOQOL scale show an initially steep incline and then a gradual stabilization of quality of life across four dimensions, including Physical Health, Psychological, Social Relations and Environment (see Table 7). The physical health of residents seemed to have been improved significantly **(p < .042)**, as well as the impact on social relations **(p < .02)**. It can also be observed that quality of life for most residents declined after the first month, pointing perhaps to initial adjustment issues with the transition. Then it seems there was a visible trend that reflected regarding their new situation, which then gradually stabilized over the remaining three months (see Table 6).

**Table 6:** Scores on QOL of the 11 residents measured six times over a period of six months (with month 4 missing).

Scale	1	SD	2	SD	3	SD	4	5	SD	6	SD
**Physical Health**	37.7	23.1	38.9	22.2	69.2	10.8	NA	53.5	5.17	54.3	12.1
**Psychological**	38.7	23.7	25.6	25.0	50	29	NA	43.6	5.2	54.3	17.1
**Social Relations**	26.9	21.8	26.2	28.5	47.6	23.6	NA	25	2.6	41.6	2.8
**Environment**	44.3	24.5	29.4	25.8	67.6	14.7	NA	41	8.62	58.3	14.8

Regarding the adaptation to the new environment, residents mentioned experiencing several differences between their new way of living versus ECRC (see Table 7).

**Table 7:** Residents’ reflections on how they perceive the SH project during the stabilization phase.

Intrusion of privacy	Some residents perceived their privacy to be less intruded upon Residents felt comfortable with fewer people staying with them There was less chaos with regard to their personal belongings There was a sense of personal space in the house
Crowding and noise	Fewer people living together makes a huge difference and makes residents feel more comfortable Their home had a quiet environment
Routine in an institution	No strict regime is enforced in the SH facility, which gives a feeling of home
One- to-one attention	There is more focused attention in the SH facility
Triggers	Residents said there were more triggers in the ECRC (*boredom*, *frustration*) provoking painful feelings than in the supported housing
Episodes of disturbed behaviour	Transition to the SH supported housing facility led to a decline in the number of incidents
Profile of residents	Residents with an intellectual disability, behavioural and personality issues had far more adjustment issues than the others

## Discussion

### Purpose of housing intervention in the context of homelessness population

‘Home’ or ‘family’ is an important place for identity formation, and integral to how behaviour becomes normalized and embedded in one’s personality. When people lose their home, they are not just homeless, but are equally at risk of losing an important frame of reference for how to function in the world. To be ‘home’ is not necessarily to be located in a particular geographical space, but to be at ‘home’ with oneself and with others. To understand the complexities of recovery processes for people with SMI, is to appreciate how their life histories are often fraught with trauma, stigma and abandonment. To rebuild their sense of dignity requires an environment for them to (re)-experience the organic evolution of a family. This is also emphasized by Wright & Kloos [37], who show that micro-level living contexts, including one’s experience of family, strongly affect wellbeing outcomes in persons with SMI and should receive adequate attention in recovery processes. Similarly, Adair, Kopp & Distasio [38] found in their work with homeless individuals with SMI that the aspects of the ‘unit’ (e.g. the type of room and how it is shared with roommates) is experienced as most valuable.

During the stability phase of this current SH project, residents in this study also shared what it meant for them to be part of ‘natural’ family setting and to experience both more privacy and togetherness (sharing a home) in their current living situation. Residents expressed various parameters of improved quality of life, by experiencing more rest, silence, attention and less intrusion in the SH facilities. This sense of being at home was translated into a greater sense of responsibility towards themselves and others. Indeed, as emphasized by Wong & Solomon [39], people with SMI are likely to assume roles as participating members of a community when provided such an opportunity and adequate support. Being able to call a place your home is therefore seen as one of the hallmarks of recovery and being well [40, 41, 42], which is also confirmed by this study.

### Shifts in operation and care in SH housing

Changing from an institutional living arrangement to a non-institutional environment had important implications regarding the functioning of the existing operating teams and guidelines. The current project emerged from the need to move some residents of The Banyan out of the ECRC, and provide support to them as part of the community. While this was a stand-alone project, it is clear that there is a wider need to support homeless individuals with mental illnesses in many parts of India [43]. For scaling-up purposes, it was deemed useful to learn from this particular transition period as experienced by The Banyan.

A few important lessons can be drawn from the different development phases. The managing team learned that it is important to move towards creating a sustainable concept of SH with the stakeholders involved, including the HCWs, the SH residents and the community. In the first phase, these stakeholders were instrumental in co-creating the vision of SH by sharing their concerns, dreams and ideas. Indeed, as shown by Acharya [44], inclusive partnerships with various stakeholders can help to produce more culturally sensitive and sustainable CBMHC programmes. With regard to this study, the residents who were given a chance to move out were supported in expressing their views on the concept of ‘home’ and SH through the initial brainstorms and drawing exercises. What emerged from these preparations were their expressed fears of being ‘left behind’, but also their wish to have a home of their own, connected to dreams of the family. Some other studies confirm that in transitions to community care, people with SMI often experience fear of insufficient support once they move out, stigma, loneliness and isolation [45, 46]. The current study results show how these fears can be addressed appropriately to help residents make the move at their own pace, which are largely in line with more current views on clients as empowered co-creators of care [47, 45]. Indeed, although the term facilitative support is not found in the literature, the underlying rationale is mentioned in similar case studies, using terms like empowerment [48], viewing residents as persons rather than patients, freedom of choice, and facilitative rather than restrictive [48, 24, 49].

Similarly, in selecting the HCWs it seemed important that the operating team carefully delineate the implications of supporting individuals with SMI in a community setting, which requires a shift in professional responsibilities. Doing this at a very preliminary stage when little is yet clearly defined, was important for HCWs to feel comfortable enough to assume the change. The HCWs who eventually moved were given autonomy in this project to co-create the concept in a way that felt good to them. As such, they took much responsibility in how they cared for the residents, addressing numerous daily issues, such as social matters of hygiene, property, meditation processes, and interactions with the community. In this, the managing team provided supportive training but also deliberately provided more freedom to HCWs and residents to work through these issues. As such, during the adaptation and confrontation phase, various strategies were employed to deal with initial incidents of ambivalence among the residents, mainly involving various trips back and forth to the ECRC, as well as visits from well-known ECRC figures to the SH facilities. The HCWs played a key role here in building trust and bridging the gap during the transition period, and being sensitive to the residents’ needs for confirmation and connection. Supporting residents in their transition, also through linking them with the community, is described by Chen [50] as a core element of community-based support programmes. It is important to emphasize these shifts in roles for staff, as supporting links with people in the community is not always considered part of mental health [51]. As seen in this study, HCWs expressed their initial discontent with some of their new responsibilities, but creating boundaries was an important strategy to help the project move into a stable phase.

Finally, the selection of the housing facility itself proved to be both challenging and critical. Finding a landlord who appreciated the intentions of CBMHC and willingness to fight stigma turned out to be an uphill task. However, renting a space in which there is sufficient leeway to experiment with slowly integrating individuals with SMI is a prerequisite for SH projects to work. Positive connections with landlords can improve the residents’ experience of social integration, housing stability, rehabilitation and successful community living. In some sense, the selection of appropriate housing deserves primary attention in the first stages of the deinstitutionalization process [52].

### Implications for scaling up

Mental health services in India for those living in poverty and homelessness are largely organized around the tertiary state mental health hospitals. The district mental health programme (DMHP), the state’s community mental health initiative, operates in only 125 out of 640 districts. Even where the DMHP runs, it primarily provides psychiatric medication, with no other interventions. Overburdened public infrastructure, lack of ongoing training and poor social care integration sustains the lack of appropriate care, especially for the poor. In facilities such as The Banyan’s, while a majority choose to and are able to go back home, 10% remain in the institution. In order to implement the SH project more widely in India, it is imperative that mental hospitals broaden their perspective, and expand their network of linkages with significant community stakeholders such as block development offices, local panchayats, village health nurses, and government bodies.

History shows the health system reform through top-down measures has been largely ineffective, while bottom-up initiatives have often failed to scale up [26]. An important component for successful upscaling are linkages between various parts of a social, economic and political system. Few studies on SH report on the importance of networks, but Nelson, Stefanic & Ray [53] indicate the significance of, for instance, government officials as beneficial to supported housing. In the current project, we saw that the more hands-on responsibility of interacting with the community lies largely with the HCWs, as well as the managing team. As explained also by Chen [50], it means that for the development of sufficient insight into the residents’ status to facilitate community integration, HCWs are required to be close to the residents, and ideally to live with them most of the time.

Although this seems like a costly project, the actual cost–benefit ratio seems to be lower in the case of The Banyan in Chennai. The cost per client in an ECRC is 20,980 INR while the cost per resident in an SH facility is 10,179 INR. So, in addition to the conceptual shift in the care paradigm, there are also substantial financial benefits. Although the cost–benefit literature for SH shows diverging results, most studies report higher costs due to the intensified care in high-income countries [54, 55]. In India, salaries are relatively low, which makes this transition more economically viable. Also, cost savings in mental health care that are associated with favourable outcomes for residents may also balance out the per capita costs of developing and implementing more intense care paths [56]. Still, while implementing SH in different market conditions in India, it might be more difficult to find affordable rental spaces, affecting its efficiency in relation to financial costs. Most importantly, therefore, to allow health systems to reform positively, this study shows that not the intervention itself is the lesson, but the adaptability of a mental health institution to find solutions in the particular context of this intervention. To reform the health system for the better, perhaps we should look beyond the diffusion of SH as intervention per se, and find ways to continuously adapt to the needs (user centred) by innovative practices through integrating services [27].

### Limitations and further research

This study is unique in exploring the challenges and strategies that emerged in working towards SH in an Indian setting. The exploratory design, including a range of stakeholders who were employed during the transition period, helped to develop socially robust knowledge, derived from contextual practice. At this stage of the project, the sample size was relatively small, and more research could be done to fully grasp the long-term benefits of SH at a micro level (for the residents, the community members, etc.) as well as macro level (economic and organizational benefits). At present, The Banyan has registered interest from other partners to scale up the SH project in several other states. This will provide the researchers with the possibility to innovate further and gain more understanding of its working mechanisms.

Finally, this pilot study relied quite heavily on the ECRC as a solid platform from which to transition. While it remains unclear from this study whether SH projects can be independently developed, it seems reasonable to assume that homeless individuals with SMI could also more directly be accommodated in SH facilities. The pioneering work with lay workers by Patel et al. [12, 55] could be particularly useful here to understand how SH might function with different readily available stakeholders (including private-sector actors, for instance). It would be valuable to conduct future research on the development of SH as independently run, and so potentially different from the current context.

## Supporting information

S1 Appendix (DOCX)
