# Supplementary material for: Supported Housing as a recovery option for long-stay patients with severe mental illness in a psychiatric hospital in South India: Learning from an innovative de-hospitalization process
Source: PLoS One. 2020 Apr 9;15(4):e0230074. doi: 10.1371/journal.pone.0230074 (PMC7144972; doi:10.1371/journal.pone.0230074)
Supplement: S1 Appendix — (DOCX) [file pone.0230074.s001.docx]

APPENDICES

**1. Topics included in the in-depth interviews with SH residents*.***

*Before the transition.*

1. What do you think about the idea and concept of moving from the psychiatric hospital to the supported housing facility?
2. Would you like to see in the new space?
3. With whom would you like to go around and see the place first?
4. Would you prefer a staff or a friend to come along with you?
5. You can see the place and then come back, or would you like to stay over the weekend and explore the environment of the space?
6. What do you expect to happen if you move out? What do you hope will happen in the new SH place?
7. If you would prefer to move out, do you have any preferences regarding roommates?
8. Do you feel assured that you can come back anytime, if you dislike residing in the supported housing facility?
9. Do you experience any fears or anxiety about moving out of one facility and move into another within the Banyan services?
10. If you would like to move out, then with whom would you like to move out? Do you have any friends or preference for people so that you can move out as friends?
11. Would you like to know the processes of transition in detail?

*During the stay in SH housing*

1. What are your experiences with living here so far?
2. What is your opinion about the new facility?
3. What do you think goes better now that you live here?
4. What do you think goes worse now that you live here?
5. Are there any challenges in living here? What would you like to see changed?
6. How are you experiencing your relationships with other roommates?
7. How are you experiencing your relationships with the HCWs?
8. How are you experiencing your relationships with the neighbourhood?
9. Do you feel your social wellbeing has changed? How has it changed?
10. Do you feel your physical wellbeing has changed? How has it changed?
11. Do you have any suggestions on how to improve the SH facility?

**2.Topics included in the in-depth interviews with SH staffs (Health Care Workers) and Mental Health Professionals (Social Worker, Psychologist)**

1. What was the motivation behind SH?
2. How was the first SH home set-up? Who initiated SH and how?
3. What are the core values and principles of SH?
4. What are the requirements for SH to work? How does it operate?
5. What has SH meant for clients who moved there? What has changed in their daily living and wellbeing?
6. What have you learned in working with patients in a SH context?
7. What is the profile of staff who played a role in SH?
8. What are the challenges and benefits in implementing SH?
9. How is SH different from independent living and CGH / other residential models of The Banyan?
10. To what extent capacity building was done for staffs who started working at SH?
11. What is the relationship of the SH home with the community/neighbours?
12. What suggestions would you give a new staff who just started working in SH?
13. What needs to be improved or maintained in order to make SH a success?
